# Author Correction: SARS-CoV-2 N protein promotes NLRP3 inflammasome activation to induce hyperinflammation

**DOI:** 10.1038/s41467-021-25629-w

**Published:** 2021-08-31

**Authors:** Pan Pan, Miaomiao Shen, Zhenyang Yu, Weiwei Ge, Keli Chen, Mingfu Tian, Feng Xiao, Zhenwei Wang, Jun Wang, Yaling Jia, Wenbiao Wang, Pin Wan, Jing Zhang, Weijie Chen, Zhiwei Lei, Xin Chen, Zhen Luo, Qiwei Zhang, Meng Xu, Geng Li, Yongkui Li, Jianguo Wu

**Affiliations:** 1 The First Affiliated Hospital of Jinan University, Guangzhou, China; 2 Guangdong Provincial Key Laboratory of Virology, Institute of Medical Microbiology, Jinan University, Guangzhou, China; 3 State Key Laboratory of Virology, College of Life Sciences, Wuhan University, Wuhan, China; 4 The Affiliated ShunDe Hospital of Jinan University, Foshan, China; 5 Foshan Institute of Medical Microbiology, Foshan, China

**Keywords:** Infection, Inflammasome, Viral host response

Correction to: *Nature Communications* 10.1038/s41467-021-25015-6, published online 02 August 2021.

The original version of this Article omitted the following from the Acknowledgements:

Science & Technology Planning Project of Guangdong Province Office of Education (2018KCXTD007, 2021XK16), Special project for the prevention and control of new crown pneumonia in Guangdong colleges and universities (2020KZDZX1060).

This has now been corrected in both the PDF and HTML versions of the Article.

